# An Empirical Study on the Satisfaction of Rural Leisure Tourism Tourists Based on IPA Method

**DOI:** 10.1155/2022/7113456

**Published:** 2022-08-03

**Authors:** Xiaoyi Wang, Linna Wang, Tianjiao Niu, Mei Song

**Affiliations:** ^1^Qinhuangdao Vocational and Technical College, No. 90, Lianfengbei Road Beidaihe District, Qinhuangdao, Hebei 066100, China; ^2^Langfang Normal University, No. 100 Aiminxidao, Langfang, Hebei 065000, China; ^3^Jeonju University, 303, Cheonjam-Ro, Wansan-Gu, Jeollabuk-Do 55069, Republic of Korea

## Abstract

In recent years, under the background of the national development strategy of rural revitalization, rural tourism has become a new way of tourism. With the upsurge of rural tourism, there has also been a wave of “homestay fever” across the country. Country house tourism is a personalized rural tourism method that has emerged with the development of our country's tourism industry in recent years. However, in the context of the surge in the number of homestays in rural tourism destinations, homogeneous competition, simple replication, low-price competition, and other quality development problems have become increasingly prominent. In order to realize the high-quality development of country houses and meet the differentiated needs of customers, it is necessary to explore the dimensions of tourists' perceived value of country houses from the perspective of tourists, to study the gap between tourists' expectations and actual perceptions, and from the perspective of tourists' value perception to make suggestions for improvement. This paper takes the theory of customer perceived value as the theoretical basis, and on the basis of referring to the relevant literature, combined with the actual situation of the homestay in Luci Village, to construct an index system of the perceived value of tourists in the country house. Use IPA (Importance-Performance Analysis) to identify the difference between the actual performance and expected importance of tourists' perceived value, use questionnaires and statistical software to analyze the difference in guest satisfaction and importance, and put forward key improvement goals to improve the quality of homestay development in the future. The effectiveness of the method proposed in this paper is verified through the case of a country house in Luci Village, Tonglu County.

## 1. Introduction

Rural tourism refers to a form of tourism that integrates sightseeing, excursions, entertainment, leisure, experience, vacation, and shopping based on the rural spatial environment, taking the unique production lifestyle, folk customs, rural scenery, rural residence, and rural culture as the object, using the differences between urban and rural areas to plan and design and combine products [1]. Through the study of the satisfaction of tourists in rural tourism, the problems existing in the process of rural tourism can be found in a timely manner, which has certain practical significance for improving the satisfaction of rural tourism tourists. At present, the domestic research on the satisfaction of tourists in tourist destinations has been relatively rich. The research content mainly focuses on the formation mechanism of satisfaction, influencing factors, evaluation models, and so on. Among them, the evaluation of satisfaction is the focus of domestic scholars' research, and scholars use the gray correlation method and importance by constructing a satisfaction evaluation index system. A large number of empirical studies have been conducted, such as the expressive analysis method (IPA) [2], the fuzzy comprehensive evaluation method [3], and the structural equation model [4]. In terms of research content, scholars explored the satisfaction of tourists such as different types of scenic spots, catering, ice and snow tourism, etc., and the choice of case places is also more diverse, which is also involved in various ethnic minority areas in China. Unfortunately, there are relatively few studies on the evaluation of tourist satisfaction in rural tourist destinations. In order to make up for the shortcomings in this regard, this paper takes the Siziwangqi as an example and uses factor analysis and IPA method to study the satisfaction of tourists in rural tourism areas, in order to provide a reference for improving the satisfaction of tourists in Siziwangqi rural tourism [5].

In recent years, with the strong support of national policies, country houses have become investment hotspots in the development of rural tourism. With the surge in the number of homestays, the quality of homestays has become increasingly prominent. Simple copying and low-price competition have become the quality pain of country houses, and the development of country houses is facing a transition period from quantitative development to quality development, which is also a key issue for the homestay industry [6].

Rural tourism occupies an important position in the modern tourism system, and the addition and improvement of the existing production structure of rural agriculture is conducive to the solution of the “three rural” problems. The development of rural tourism can improve the lives of farmers, provide opportunities for rural employment, and add impetus to rural revitalization and the development of other related industries. Academia has carried out a lot of research on the concept definition of rural tourism, influencing factors, development and management of destinations, tourism planning, dynamic mechanisms, and sustainable development.

On the whole, China's country houses are still in the production-oriented primary development stage, the market demand for homestays is insufficient, and there have been development problems such as similarity in hardware and services, design plagiarism, and further intensification of competition. In the context of the experience economy era, tourists have higher and higher requirements for experience and personalized, and in-depth understanding of the country house experience perception of Chinese consumers is a key problem in solving the development of homestays, and it is also a major basis for guiding the high-quality development of China's country houses in the future, and it is urgent to carry out research [7].

The research on the perceived value of tourists in the field of tourism involves heritage tourism, hotel industry, fast food industry, festival tourism, inbound tourism, ancient village tourism, etc. The measurement dimension of perceived value has also shifted from the initial single-dimensional measurement to the multidimensional measurement. However, research on the perceived value of tourists in the context of country houses has not yet been fully developed. Therefore, this study provides a new theoretical perspective on the quality improvement of country houses using the theory of customer perception value [8].

Reviewing the literature, it is found that, among the existing research results, most of the research on the perceived value of tourists in specific cities or large tourist destinations is studied, while the research on the perceived value of tourists is relatively weak in terms of country houses on a small scale. Therefore, this paper takes the Luci Village in Tonglu County as an example, conducts an empirical analysis of the perceived value of homestay tourists, and uses the IPA method to study the satisfaction of country house tourists with all dimensions of perceived value and then provides a scientific theoretical basis and experience for the improvement of the perceived value of country houses [9].

## 2. State of the Art

1. Research status of foreign tourism marketing: The research on tourism marketing in Western developed countries started earlier, and a relatively mature theoretical system has been formed. Some experts and scholars believe that the tourism resources of various countries are different, and this difference will lead to obvious differences in the level and characteristics of tourism and development in various countries. After statistical analysis, it is understood that countries with roughly the same level of economic development also show many common points in the development of the tourism market. However, it should be noted that the closer the economic development level of the two countries, the higher the overlap of residents' tourism needs, which in turn provides a good opportunity for tourism cooperation between the two countries. It can be seen that the level of national economic development is closely related to the development of the entire tourism industry [10].
2. Nasir (2020) found that many factors such as national economic development and price level can affect the development of domestic tourism to varying degrees. Based on this, in the process of supporting tourism, the state should choose the above elements as the entry point to achieve the orderly and healthy development of tourism [11].
3. Zhang et al. calculate the development potential of regional tourism through mathematical statistics, which can reasonably predict the future development of regional tourism. By measuring the tourism development potential of countries with different economic development levels, it provides a new basis for the tourism development of countries with different economic development levels [12].
4. Purwanto et al. conducted a survey of tourism marketing mainly from the perspective of tourist characteristics and found that tourists pay more attention to the geographical characteristics of tourist areas. In recent years, people have gradually adopted scientific research methods to study the impact of high technology on the development of tourism, and through surveys, it is found that tourists tend to consider whether the economy of tourist destinations is developed when choosing tourist destinations and tend to go to areas with better economic development [13].
5. The current status of domestic research on tourism marketing.
6. At present, there is not much research on tourism marketing in China, mainly focusing on the formulation of marketing tools, marketing channels, and marketing strategies.
7. Liu et al. said that when tourism marketing matches the psychological characteristics of individuals, marketing effectiveness will be greatly improved. Therefore, in the process of optimizing tourism marketing, the marketing content is more likely to be popular, so as to better attract tourists. Tourism marketing essentially belongs to the same category of marketing, so it is also in line with the 4P theory of marketing [14]; Hong et al. said that, with the improvement of people's quality of life, the demand for tourism has also increased significantly, so it provides an important impetus for the development of the entire tourism market. In this case, the development of the tourism industry should adopt the method of customized marketing and experience marketing for tourism marketing, so that tourism marketing can better meet the needs of customers and achieve the purpose of attracting tourists [15]; Ashton studied the marketing environment for the development of the tourism market and found that the influencing factors that control the development of the tourism industry provide certain support and help for the development of the entire tourism industry [16].

Domestic scholar Azhar et al. has carried out a systematic analysis of tourism marketing channels as the focus. He stressed that the emergence and application of new media has further broadened tourism marketing channels, so in the development process of tourism market, attention should be paid to the rational use of emerging media. He said that relying on new media such as WeChat platform and Weibo to carry out tourism marketing work is conducive to expanding the international influence of the tourism market [17].

Johann and Ghose said that, in the current context, the most in line with the form of the tourism market is experiential tourism marketing. Experience marketing can enhance the tourist experience and increase satisfaction of tourists by meeting the service needs of tourists to the greatest extent possible [18], and Chinese tourism expert Kuo et al. focused her research on tourism marketing strategies. They said that, in the development of the tourism industry, it should choose a tourism marketing strategy that is compatible with the current development of the country as much as possible. In addition, for different tourism regions and target groups, there should also be a comprehensive consideration. In order to promote the domestic tourism market, the government should strengthen publicity and enhance the international visibility of the domestic tourism market [19].

In summary, although the current academic research on the digitalization of rural tourism has achieved fruitful results, the existing research pays less attention to the service quality, effect evaluation, and tourist perception of the digital development of rural tourism. In addition, the research is mainly conceived from the perspective of governments and enterprises, and the lack of research related to the digital development of rural tourism from the perspective of tourists ignores the main differences and correlation effects to a certain extent [20].

## 3. Methodology

**Figure 1:**
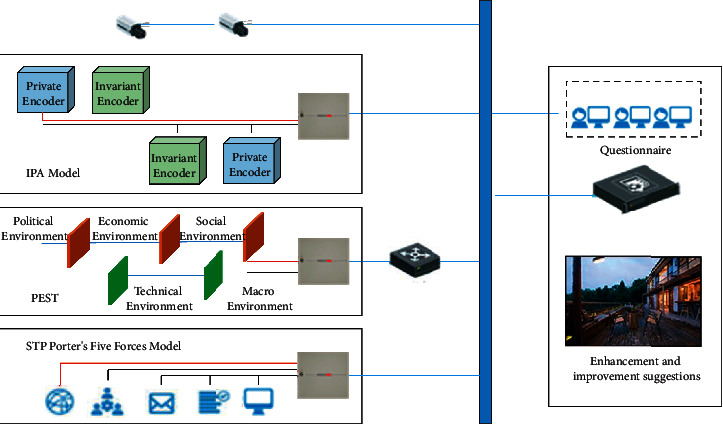
The process of the homestay operation quality improvement plan.

The concepts involved in this article include the concepts related to characteristic towns, tourism motivation, and tourism marketing concepts. The theoretical foundations that will be applied include PEST analysis, Porter's five forces model, STP target market strategy, and 4P combined marketing. Therefore, this chapter mainly expounds the above concepts and theoretical basis and introduces the program flow of the full text, as shown in Figure 1.

### 3.1. Overview of the IPA Model

In IPA (Importance-Performance Analysis) method, importance is the user's attention to products or services and other attributes, and performance refers to the user's measurement of the actual performance of the product or service. The IPA method requires respondents to evaluate each assessment element in terms of both importance and performance of the measures of the specified respondents. When used for satisfaction measurement, performance is a satisfaction evaluation.

**Figure 2:**
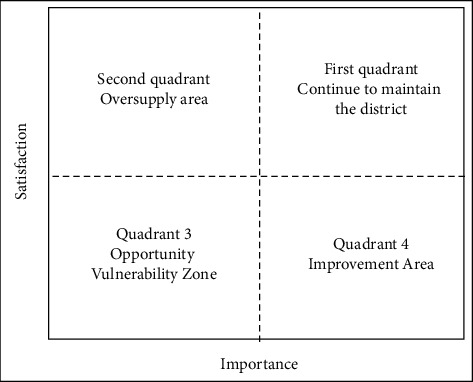
IPA plot.

The IPA method, proposed in 1977 by Martilla and James in the study of the properties of industrial products, has the basic idea of measuring customer ultimate satisfaction by comparing customer expectations for products and actual performance of products. The main method of establishing the IPA model is to list the customer's attention (importance) to the product as the horizontal axis (*X* axis) and the actual performance (satisfaction) of the product as the vertical axis (*Y* axis), construct a coordinate system, and use the total average of the customer's evaluation of the importance and performance of each attribute of the product as the partition point of the *X* axis and the *Y* axis, divide the space into 4 quadrants, and fill in the corresponding positions with the actual scores of the importance and performance of each factor The main principle is to establish a four-quadrant matrix according to the importance of evaluation indicators and the level of satisfaction, compare the importance and performance of different dimensions, and provide support for decision-makers' decision-making. Among them, from the top right to the bottom right, in the first quadrant: the importance and performance are relatively high, the factors in this area occupy the main position, and the enterprise should continue to maintain its advantages in the future operation, in order to continue to maintain the zone; in second quadrant: there is low importance and high performance; although the importance of this factor is low, its resource optimization degree is better; it belongs to the secondary advantage of the location; in the near future there is no need to spend too much energy; in third quadrant: there is low importance and performance, secondary disadvantage, not priority development; in fourth quadrant: there is high importance and low performance; the region is very valued but the performance is not satisfactory; there are many factor disadvantages; enterprises should improve this in the process of development; it is the focus of improvement areas. This is shown in Figure 2.

### 3.2. Introduction to PEST

PEST analysis is the basic tool of strategic external environment analysis, which grasps the macroenvironment from the perspective of politics, economics, society, and technology or four aspects of factor analysis from the overall perspective and evaluates the impact of these factors on corporate strategic objectives and strategy formulation.

The political environment mainly refers to the national macro political environment and policy content, and a stable and harmonious political environment has a major impetus for industrial development;

The economic environment refers to all economic conditions that occur during the operation of the enterprise, such as the national economic development trend and the level of national economic development. The economic environment of the country is closely related to the operation and development of the enterprise; in particular it can have a direct impact on the strategic planning and marketing strategy of the enterprise.

The social environment mainly reflects the social institutions of a country or region, educational status, customs and culture, etc. It should be noted that the above elements affect the determination of the business strategy of the enterprise to varying degrees.

The technological environment refers to the technological capabilities and technological development trends of a country or region. The improvement of technical capabilities requires enterprises to transform and upgrade, emphasizing the research and development of new technologies, so that the technical level can maintain the mainstream or even the leading level.

Macroenvironmental factors can have a direct effect on the strategic planning of enterprises. But usually, the impact of the enterprise on it is not obvious. Considering the particularity of the macroenvironment, enterprises are required to attach importance to the continuous adjustment of their own strategic goals to ensure that the development of enterprises is compatible with social development trends.

### 3.3. Introduction to the Porter Five Forces' Model

**Figure 3:**
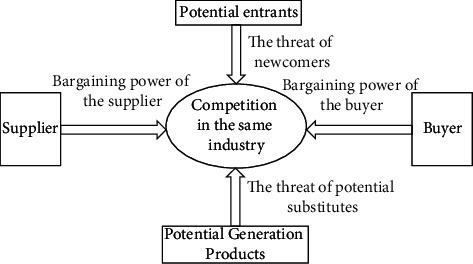
Porter's five-force model.

The Porter Five Forces' model was originally proposed by Porter. From the composition of the model, the upstream and downstream dimensions of the product are mainly divided into the bargaining power of upstream suppliers and the bargaining power of downstream buyers; the internal dimension of the industry is the competitiveness of the industry; and the external dimension of the industry includes product substitution and potential competitors. Therefore, the model usually uses the above aspects as an entry point to measure and evaluate the competitive situation of the target industry. Through this model, it can be understood that, due to the differences in the industry, there are also differences in the degree of influence of the above factors. Therefore, enterprises should pay attention to the analysis of competitive strategies. For its model structure you can refer to Figure 3.

### 3.4. STP Target Market Strategy

The concept of market segmentation was first defined and illustrated by Wendell Smith in the United States in the middle of the last century. With the further deepening of scholars' market research, Philip Kotler supplemented and improved the theory on the original basis and finally constructed the STP target market strategy theory. STP is the three elements of marketing strategy in marketing; in modern marketing theory, market segmentation, market positioning, and market positioning are the three core elements that constitute the marketing strategy of enterprises, known as STP marketing.

**Figure 4:**
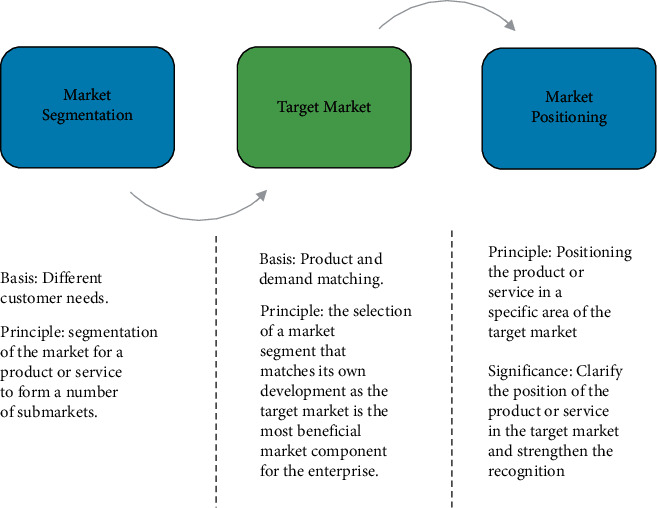
STP target market strategy implementation steps.

The implementation of STP theory first needs to distinguish between different market segments. After the market segmentation, the enterprise should comprehensively consider various factors and finally clarify the target market that meets its own requirements; subsequently, the enterprise criticizes the needs of the target customers according to the good resources and capabilities it has, and on this basis, it carries out targeted marketing activities to further deepen the understanding of the target group of the enterprise to achieve the purpose of market positioning. The specific process can refer to Figure 4.

### 3.5. 4P Combo Marketing

**Figure 5:**
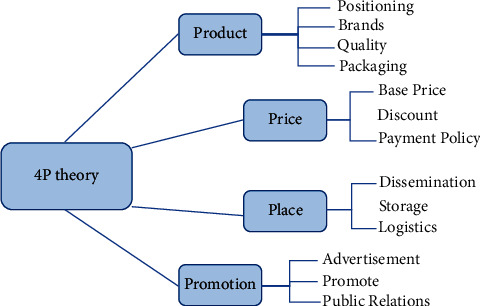
4P combined marketing theory.

4P Marketing Theory was originally formed in the 1960s and is an extension of the original marketing mix theory. As early as the middle of the last century, Neil Boden specifically elaborated and explained the concept of “marketing mix.” He stressed that market demand depends heavily on “marketing variables” or “marketing elements.” Specifically, the 4P represents Product, Price, Place, and Promotion, as shown in Figure 5.

### 3.6. Principle of Support Vector Classifier

In this paper, the evaluation of TSTD is summarized as a multiclass classification problem on the basis of reconstructing the existing tourist satisfaction evaluation index system of tourist destinations. According to the characteristics of the influencing factors of tourists in the tourist destination, the specific evaluation index is quantified by the method of normalization after questionnaire survey, and then it is used as the input sample of SVM to establish a tourist destination tourist satisfaction evaluation based on support vector machine. The test results show that the evaluation method is feasible. The research of support vector machine was originally proposed for the two-class linear separable problem in pattern recognition, and its core technology is to construct the optimal hyperplane.

**Figure 6:**
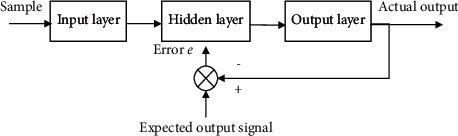
SVM optimal classification surface.

The basic idea of SVM can be illustrated by the two-dimensional situation shown in Figure 6: the solid points and hollow points in the figure represent two types of samples, H is the classification line, and H1 and H2 are the closest and parallel to the classification line passing through the two types of samples. The distance between classification lines is called classification interval. The so-called optimal classification line requires that the classification line can not only separate the two classes correctly (the training error rate is 0), but also maximize the classification interval. If H satisfies the condition of the optimal hyperplane, the training samples on H1 and H2 are called support vector machines.

The mathematical description is as follows: Assume that there are training samples that {*x*_*i*_, *y*_*i*_}, *i*=1,2,…, *n*, *x*_*i*_ ∈ *R*^*n*^, *y*_*i*_ ∈ {−1, +1} are class labels. In the case of linear separability, there will be a hyperplane that completely separates the two types of samples. The classification hyperplane equation is

(1) $$\begin{array}{c} \omega \cdot x_{i}+b=0, \end{array}$$

where *ω* is the weight vector (the normal vector of the hyperplane) *ω* · *x*_*i*_ representing the inner product of the vector *ω* ∈ *R*^*n*^ and *x*_*i*_ ∈ *R*^*n*^; *b* is the bias, also known as the classification threshold. The samples are classified as follows:

(2) $$\begin{array}{c} \omega \cdot x_{i}+h\geq 0\left(y_{i}=+1\right),\\ \omega \cdot x_{i}+b\leq 0\left(y_{i}=-1\right). \end{array}$$

Solving the optimal hyperplane is to find the optimal value of *ω* and *b* for a given training sample to minimize the weight cost function, namely:

(3) $$\begin{array}{c} \min\phi \left(x\right)=\frac{1}{2}{\left|\omega \right|}^{2},\\ \text{s}.\text{t}.\;y_{i}\left(\omega \cdot x_{i}+b\right)\geq 0\;i=1,2,\ldots ,n. \end{array}$$

When the training samples are linearly inseparable, a nonnegative slack variable needs to be introduced, and the hyperplane optimization problem is

(4) $$\begin{array}{c} \min\phi \left(x\right)=\frac{1}{2}{\left|\omega \right|}^{2}+C\sum_{i=1}^{n}\xi_{i},\\ \text{s}.\text{t}.\;y_{i}\left(\omega \cdot x_{i}+b\right)\geq 1-\xi_{i}\xi_{p}\geq 0. \end{array}$$

Using the Lagrange function, the original linearly separable optimization problem can be reduced to a dual problem:

(5) $$\begin{array}{c} \max W\left(\alpha \right)=\sum_{i=1}^{n}\alpha_{i}-\frac{1}{2}\sum_{i=1}^{n}\sum_{j=1}^{n}\alpha_{i}\alpha_{j}y_{i}y_{j}\left(x_{i}x_{j}\right),\\ \text{s}.\text{t}.\;\sum_{i=1}^{n}y_{i}\alpha_{i}=0\alpha_{p}\geq 0. \end{array}$$

By solving the above quadratic programming problem, the corresponding optimal *a* and *b* threshold is obtained. Only the corresponding samples determine the classification result. These samples are called support vectors. The classification decision function is

(6) $$\begin{array}{c} f\left(x\right)=\mathrm{sgn}\left[\sum_{i=1}^{n}y_{i}\alpha_{i}^{*}\left(x_{i}x_{j}\right)+b^{*}\right]. \end{array}$$

For linear inseparable problems, a nonlinear mapping function can be introduced to map the samples to a certain high-dimensional space and convert it into a linear classification problem in the attribute space. This nonmapping function is called a kernel function. According to the Mercer condition, the linear classification of a nonlinear transformation can be realized by using different inner product functions in the optimal classification surface. The corresponding classification decision function is

(7) $$\begin{array}{c} f\left(x\right)=\mathrm{sgn}\left[\sum_{i=1}^{n}y_{i}\alpha_{i}^{*}\left(x_{i}x_{j}\right)+b^{*}\right]. \end{array}$$

The basic idea of applying the SVM model for classification can be summarized as follows: first, map the input vector to a feature space, and then find the optimal linear dividing line in the feature space; that is, construct a hyperplane that can separate the two classes so that the two classes can be separated correctly. Since the support vector machine theory only considers the dot product operation in the high-dimensional feature space *K*(*x*_*i*_, *x*_*j*_)=*φ*(*x*_*i*_) · *φ*(*x*_*j*_), instead of using the function *φ* directly, it cleverly solves the problem that *ω* cannot be expressed explicitly because the mapping function *φ* is unknown. The symmetric function that satisfies the Mercer condition is called the kernel *K*(*x*_*i*_, *x*_*j*_) function mentioned above. Commonly used kernel functions include polynomial kernel function, Sigmoid kernel function, and radial basis function kernel function. In this paper, the radial basis function kernel function is used:

(8) $$\begin{array}{c} K\left(x_{i},x_{j}\right)=\exp\left(\frac{-{\left|x_{i}-x_{j}\right|}^{2}}{\sigma^{2}}\right). \end{array}$$

## 4. Result Analysis and Discussion

### 4.1. Satisfaction Analysis of Rural Leisure Tourism Tourists

A total of 318 effective respondents in this study were statistically described from gender, age, education, occupation, monthly income, place of residence, number of stays, etc., and the specific results were shown in Table 1 as shown. From the perspective of gender distribution, women accounted for 67.3% and men accounted for 32.7%; from the perspective of age characteristics, the sample age group was mainly concentrated between 18 and 40 years, accounting for 79.2% of the total, indicating that the main young and middle-aged people stay in the country house; from the perspective of education level, the education of the interviewed subjects was mainly concentrated in this specialty, accounting for 77.04% of the total, indicating that the education level of consumers who chose homestays was generally better; from the perspective of occupational composition distribution, most of the samples were students and employees of enterprises and institutions, accounting for 45.6% and 26.7% of the total, respectively, indicating that most of the respondents had stable incomes; from the perspective of monthly income characteristics, ranging from 1,000 yuan to 8,000 yuan, because the daily house price of Tonglu Luci Homestay was in the range of 300–800 yuan, the spending power requirements for customers were low.

### 4.2. Descriptive Statistical Analysis of Variable Data

Descriptive statistical analysis can roughly indicate the degree of dispersion of data, the trend of concentrated distribution, and so on. Descriptive statistical analysis of questionnaire data include sample means, standard deviations, skewness, and kurtosis of perceived value satisfaction and important metrics, thus judging the basic level of the questions in the scale and the distribution of the data presentation. When the absolute value of the skewness of the measurement index is less than 3 and the absolute value of the kurtosis is less than 8, it means that the sample follows the normal distribution.

According to the analysis results of Table 2, the survey data are basically in line with the normal distribution. The average value of each item of social value is between 2.5 and 3.0, indicating that consumers have a general experience of social perception value of homestays and fail to fully meet their sense of taste superiority and social status. The average value of each item on environmental value is between 3.5 and 5.0, indicating that consumers are in a relatively satisfied state with the experience of perceived value of the homestay environment. The average value of each item of convenience value is between 3.5 and 5.0, indicating that consumers are also in a relatively satisfied state with the experience of convenience value of homestays. The average value of each item on service value is between 2.5 and 3.0, indicating that consumers' service value experience of homestays has not been fully satisfied. The average value of each item of leisure value fluctuated around 3.0; in particular the average value of the third question item on the interaction and fun of the activity was 2.2, indicating that tourists were dissatisfied with the satisfaction of the leisure value of the homestay. The average value of each item of emotional value fluctuates around 4.0, indicating that the satisfaction of tourists with emotional value is very high.

### 4.3. Reliability Analysis

Reliability analysis is a test of the reliability of questionnaire findings. The commonly used coefficient is the reliability factor *α*, which is generally between 0 and 1, and the closer the *α* is to 1, the more reliable the survey results are. In general, the reliability factor *α* is high if it is above 0.8, the reliability is good if the *α* value is between 0.7 and 0.8, the reliability of the survey is acceptable if the *α* value is between 0.6 and 0.7, and if the *α* value is below 0.6, the survey result should be abandoned. According to the variable reliability analysis table, the satisfaction questionnaire was obtained with a Cronbach's *α* coefficient of 0.934 and a Cronbach's *α* coefficient of 0.925, both greater than 0.8, indicating that the questionnaire scale of the study has good reliability. The results are shown in Table 3.

According to the perceptual value satisfaction scale, Cronbach's *α* values of social value, environmental value, convenience value, service value, leisure value, and emotional value are 0.879, 0.821, 0.817, 0.908, and 0.810, respectively; that is, Cronbach's *α* values of the first five variables are greater than 0.80, indicating that the reliability is very good, and the last variable has a sentiment value of 0.775, indicating that the variable has a good reliability; that is, all variables have a high reliability. At the same time, CITC is also greater than 0.3, which meets the requirements of the reference standard and can be analyzed for subsequent data, as seen in Table 4.

From the importance data of actual performance, Cronbach's *α* values of social value, environmental value, convenience value, service value, leisure value, and emotional value are 0.848, 0.842, 0.853, and 0.881, respectively; that is, Cronbach's *α* values of the first four variables are greater than 0.80, indicating that the reliability is very good, and the last two variables, leisure value and emotional value, are 0.799 and 0.758, respectively, indicating that the reliability of the variables is better. That is, all variables have a high degree of confidence. At the same time, CITC is also greater than 0.5, which meets the requirements of the reference standard and can be analyzed for subsequent data; see Table 5.

### 4.4. Validity Analysis

Validity analysis is the measurement of the correctness of the questionnaire data. In this study, factor analysis methods will be used to test the validity of the measurements. The KBO sample adequacy measure and the Bartlett sphere test are used to determine whether the data meets the criteria of factor analysis. If the KMO is above 0.90, it means that the scale validity is very good; if the KMO is between 0.7 and 0.9, it means that the validity is acceptable; if the KMO is between 0.5 and 0.7, it means that the validity is average; if the KMO is below 0.5, it means that the validity is not acceptable, and some items of the scale need to be modified. In addition, when the statistical significance probability of the Bartlett spherical degree test is less than or equal to the significance level, factor analysis can be done; see Table 4.

As can be seen from the above table: KMO is 0.930, greater than 0.6, which meets the premise requirements of factor analysis, which means that the data can be used for factor analysis studies. And the data passed the Bartlett spherical degree test (*p* < 0.05), indicating that the study data are suitable for factor analysis.

The following table analyzes the factor extraction situation and the amount of factor extraction information, which can be seen from Tables 4–6: The factor analysis extracts a total of 6 factors, the characteristic root values are greater than 1, and the variance interpretation rate after rotation of these 6 factors is 16.926%, 12.213%, 11.147%, 11.083%, 9.241%, and 8.654%, and the cumulative variance interpretation rate after rotation is 69.264%, respectively, greater than 60%, indicating that the extracted factors can well explain the information contained in the 24 question items.

The data for this study were rotated using the maximum variance rotation method (varimax) to find the correspondence between factors and study items. The above table shows the information extraction of factors for research items, as well as the correspondence between factors and research items; it can be seen from the above table that the common value corresponding to all study items is higher than 0.5, which means that there is a strong correlation between the study items and factors, and the factors can effectively extract information. After ensuring that the factor can extract most of the information of the research item, and then analyzing the correspondence between the factor and the study item, the specific 6-factor structure of the factor corresponding factor relationship can be obtained, which is consistent with the theoretical design, so the questionnaire has good validity.

## 5. Conclusion

Taking the Luci Village in Tonglu County, Zhejiang Province, as an example, this article studies the differences in the importance and satisfaction of tourists' perceived value dimension from the perspective of perceived value and draws the following conclusions through case analysis, research design, survey visits, sample analysis, data analysis, and other operations.

The satisfaction and importance of social value in this study are lower than 3, indicating that the satisfaction and importance of this dimension are low, which is a weak area of opportunity; from the perspective of tourists, tourists have lower expectations for the social superiority experienced by staying in homestays, and at the same time, the actual management of homestay managers does not attach importance to social value, so the satisfaction of tourists with social value perception is also low.

The importance of homestay tourists to social value is low, mainly due to the following aspects: First, the homestay house price in Luzi Village ranges from 300 to 800 yuan, catering to the spending power of low-end consumer groups. Tourists who choose Tonglu Luci Village Homestay are mostly students and young office workers; due to the limitations of economic strength, this part of the group lacks high spending power, but they are enthusiastic about exploring new things, willing to try and experience various types of country houses, but the expectation that staying in the homestay can enhance the sense of social superiority is low. According to the satisfaction analysis of each question, the average satisfaction value of the four items of social value is less than 3 points, so the satisfaction of homestay tourists with social value is low. In terms of space design, the difference between the homestay and the hotel is that the homestay will design a relatively large leisure space, so that the customers who stay in the homestay can relax in the leisure space during the rest time; this public space provides a platform for mutual communication between the host and the guest, and the customer can have the opportunity to know strange friends from all over the world. Moreover, some homestay owners will also organize customers to participate in some leisure activities, such as tea-themed homestay owners who will use their tea art skills to invite customers to come to taste tea and talk freely, which naturally provides an opportunity for homestay guests to communicate with each other and get to know each other. However, under the influence of the epidemic, fewer people are traveling, and the number of tourists staying in homestays has decreased, and the probability of homestay guests meeting new friends has naturally decreased. As a result, homestay visitors are also less satisfied with the dimension of social value. As the “social value dimension” of the opportunity disadvantage area, although there is no need to take any action strategy at present, in the long run, due to the low satisfaction of tourists, homestay operators should strengthen tracking and observation.

Based on the above conclusions, this article makes the following recommendations:

1. Adhering to the beautiful natural scenery of the ecological county is the biggest advantage of Tonglu, so Tonglu put forward “one major goal and three major strategies.” One of the major goals is to build a beautiful Chinese Tonglu sample with beautiful mountains and rivers and strong Minfu County, and the three major strategies include ecological counties, strong industrial counties, and national Xing counties. The premise of everything is to protect the natural ecology and promote the sustainable development of global tourism.
2. Pay attention to cultural accumulation.
3. Landscape is the clothing; culture is the core. Tonglu is a small town in Jiangnan on the banks of the Fuchun River, which has accumulated a profound history and culture. The government insists on protecting cultural relics as a breakthrough in the entire Tonglu County and surrounding areas, repairs and transforms at the same time, combines the protection of natural landscapes with cultural excavation, pays attention to the protection of traditional buildings and folk customs, exerts cultural charm, and gives tourists a real cultural and beauty experience. The integration of tourism industry and cultural industry is an important part of the development of rural tourism, combining history and culture with modern culture, not only to dig deep into the essence of ancient culture, but also to focus on cultural innovation, promote cultural and creative projects, inherit history, and develop the future.
4. Create tourism characteristics.
5. In order to meet the market demand, Tonglu has deeply developed leisure vacation experience tourism projects, accelerated the integration of elements such as “small stay,” “shopping,” “catering,” “play,” and “leisure,” vigorously promoted the combination of tourism and industrial development, opened up health and leisure tourism supported by traditional Chinese medicine, sports health tourism, and life and health tourism based on the national football training base, and made every effort to create a characteristic tourism county. Rural tourism must have characteristics; otherwise it will enter vicious competition and cannot achieve sustainable development.

## Figures and Tables

**Table 1:** Demographic analysis of samples.

Individual characteristics	Options	Frequency	Effective percentage (%)	Cumulative percentage (%)
*Gender*	Man	104	32.704	32.704
	Woman	214	67.296	100.00

*Age*	Under 18 years of age	3	0.943	0.943
	18–25 years old	176	55.346	56.289
	26–30 years old	38	11.950	68.239
	31–40 years old	38	11.950	80.189
	41–50 years old	24	7.547	87.736
	51–60 years old	32	10.063	97.799
	60 years old and above	7	2.201	100.00
	High school and below	45	14.151	14.151

*Degree*	College or undergraduate	245	77.044	91.195
	Graduate and above	28	8.805	100.0
	Student	145	45.597	45.597

*Occupation*	Corporate employees	42	13.208	58.805

**Table 2:** Descriptive statistical analysis of perceived value satisfaction data.

Dimension	Question	Number of cases statistics	Minimum statistics	Maximum statistics	Average value total measurement	Standard value statistics	Skewness total measurement	Kurtosis is measured	Kurtosis is measured	Standard error
*Social value*	A1	318	1.00	5.00	2.664	1.105	−0.010	0.137	−0.996	0.273
	A2	318	1.00	5.00	2.620	1.096	0.119	0.137	−0.766	0.273
	A3	318	1.00	5.00	2.695	1.103	−0.027	0.137	−0.855	0.273
	A4	318	1.00	5.00	2.774	1.134	−0.147	0.137	−0.936	0.273
	A5	318	1.00	5.00	3.865	1.169	−1.070	0.137	0.499	0.273

*Environmental value*	A6	318	1.00	5.00	3.811	1.144	−1.000	0.137	0.408	0.273
	A7	318	1.00	5.00	3.881	1.153	−1.107	0.137	0.577	0.273
	A8	318	1.00	5.00	3.874	1.158	−1.066	0.137	0.484	0.273
	A9	318	1.00	5.00	3.877	1.030	−0.798	0.137	0.149	0.273

*Convenience value*	A10	318	1.00	5.00	3.884	1.018	−0.865	0.137	0.393	0.273
	A11	318	1.00	5.00	4.076	0.999	−1.165	0.137	1.151	0.273
	A12	318	1.00	5.00	4.016	1.019	−1.023	0.137	0.704	0.273
	A13	318	1.00	5.00	2.833	1.168	−0.330	0.137	−1.149	0.273
	A14	318	1.00	5.00	2.862	1.162	−0.347	0.137	−1.099	0.273
	A15	318	1.00	5.00	2.877	1.176	−0.370	0.137	−1.133	0.273

*Service value*	A16	318	1.00	5.00	2.846	1.153	−0.342	0.137	−1.102	0.273
	A17	318	1.00	5.00	2.884	1.176	−0.381	0.137	−1.128	0.273
	A18	318	1.00	5.00	2.881	1.169	−0.397	0.137	−1.121	0.273
	A19	318	1.00	5.00	3.110	1.144	−0.675	0.137	−0.732	0.273

*Leisure value*	A20	318	1.00	5.00	3.104	1.148	−0.633	0.137	−0.733	0.273
	A21	318	1.00	5.00	2.201	1.094	0.526	0.137	−0.428	0.273
	A22	318	1.00	5.00	3.984	1.006	−0.997	0.137	0.662	0.273

*Emotional value*	A23	318	1.00	5.00	4.076	0.899	−0.831	0.137	0.489	0.273
	A24	318	1.00	5.00	4.000	0.995	−1.063	0.137	0.949	

**Table 3:** Variable reliability analysis.

Variable	Cronbach's *α* coefficient	The number of items
Satisfaction	0.925	24
Importance	0.925	24

**Table 4:** Satisfaction questionnaires for KMO and Bartlett's tests.

KMO value		0.930
Bartlett spherical degree test	Approximate chi-square	4003.686
	df	276
	*P* value	psD

**Table 5:** Reliability test of the perceptual value important measure.

Variable	Question	CITC	After deleting the indicator Cronbach's*α*	Overall Cronbach's *α*
*Social value importance*	B1	0.693	0.805	0.848
	B2	0.737	0.785	
	B3	0.654	0.821	
	B4	0.663	0.818	
	B5	0.645	0.813	

*Environmental value importance*	B6	0.661	0.807	0.842
	B7	0.631	0.820	
	B8	0.773	0.755	
	B9	0.690	0.815	
	B10	0.720	0.803	

*Convenience value importance*	B11	0.699	0.811	0.853
	B12	0.668	0.824	
	B13	0.352	0.862	
	B14	0.380	0.864	
	B15	0.706	0.858	

*Service value importance*	B16	0.668	0.866	0.881
	B17	0.715	0.856	
	B18	0.714	0.857	

**Table 6:** Reliability test of perceived value satisfaction scale.

Variable	Question	CITC	After deleting the indicator Cronbach's *α*	Overall Cronbach's *α*
*Social value importance*	A1	0.727	0.805	0.879
	A2	0.771	0.833	
	A3	0.737	0.846	
	A4	0.0.722	0.852	

*Environmental value importance*	A5	0.623	0.785	0.821
	A6	0.657	0.769	
	A7	0.642	0.775	

*Convenience value importance*	A8	0.653	0.771	0.817
	A9	0.678	0.752	
	A10	0.626	0.8776	
	A11	0.616	0.781	

*Service satisfaction importance*	A12	0.633	0.773	0.908
	A13	0.748	0.891	
	A14	0.732	0.893	
	A15	0.752	0.889	

## Data Availability

The labeled data set used to support the findings of this study is available from the corresponding author upon request.
